# Splenic Injury After a Colonoscopy: Threading the Scope Carefully in Heritable Connective Tissue Disorders

**DOI:** 10.7759/cureus.15444

**Published:** 2021-06-04

**Authors:** Brandon Wiggins, Cassandra Lamarche, Rohit Gupta, Smit Deliwala, Mark Minaudo

**Affiliations:** 1 Internal Medicine, Ascension Genesys Hospital, Grand Blanc, USA; 2 School of Medicine, Michigan State University College of Human Medicine, East Lansing, USA; 3 Internal Medicine/Pediatrics, Michigan State University, Flint, USA; 4 Internal Medicine, Michigan State University at Hurley Medical Center, Flint, USA; 5 Gastroenterology and Hepatology, Ascension Genesys Hospital, Grand Blanc, USA

**Keywords:** colonoscopy complications, marfan disease, connective tissue disorder, ehlers danlos, colon polypectomy, colon polypectomy

## Abstract

Colonoscopies have reduced colorectal cancer (CRC) burden in the United States, and their utility has expanded to include various diagnostic and therapeutic indications. Complications are seen in up to 1% and increase with age and polypectomy. As colonoscopies become widespread, specific populations seem to be at a much higher risk; notably patients with heritable connective tissue disorders (HCTD). As life expectancy increases, these patients undergo routine screenings and require careful peri-endoscopic care to reduce adverse outcomes. Amongst HCTD, Ehlers-Danlos syndrome (EDS) is commonly implicated, however, no reports of Marfan syndrome (MS) exist. We present a unique case of splenic injury after colonoscopy in a patient with MS. Successful outcomes require early suspicion and emergent surgical evaluation in patients with hemodynamic instability after a colonoscopy. Increased ligament laxity and bowel fragility are the most likely mechanisms. Alternative CRC strategies like fecal immunochemical test (FIT), fecal occult, Cologuard, or virtual colonography can be considered.

## Introduction

Colonoscopy is considered the primary modality for colorectal cancer (CRC) prevention. It is considered a safe and routine procedure, offering both diagnostic and therapeutic potential [1]. Currently, there are over 10 million colonoscopies performed annually in the United States. To improve the quality and safety of colonoscopy, the American Society of Gastrointestinal Endoscopists (ASGE), developed guidelines that outline quality metrics during the peri-endoscopic period for safe and effective intubations [2]. Despite this, pooled studies have revealed colonoscopy complication rates ranging between 0.2%-1% [3,4].

Amongst known complications, splenic injuries occur at a rate of 0.20 per 10,000 colonoscopies, carrying a high mortality rate [4,5]. As the volume of colonoscopies rises, splenic injuries are being reported increasingly more frequently, and risk factors such as age, female sex, splenomegaly, adhesions from prior surgeries, malignancy, sepsis, polypectomy, or ongoing anticoagulation have been parsed out [3,6,7]. The risk for mortality is most significant in the acute inpatient setting, and lack of early identification of splenic injuries can prolong hospital stay and reduce the quality of life [8]. Additional factors implicated in safety include operator experience, dexterity, sedation, hospital capabilities, and logistical challenges [3].

Marfan syndrome (MS), an autosomal dominant systemic tissue disorder, is one of the more common heritable connective tissue disorders (HCTDs) impacting 2.5 per 10,000 individuals [9]. As the life expectancy of those with HCTD increases, they are encouraged to follow the same screening protocols as non-HCTD cohorts. As a result, connective tissue disorders pose a substantial risk to gastroenterology practices for spontaneous and instrumentation-related injuries [4]. Due to the variable penetrance of HCTDs, a wide array of phenotypic presentations exist, with many remaining undiagnosed for years [10]. Additionally, we performed a systematic review for cases of splenic injury in HCTDs. Data guiding patient selection, management, and surveillance of these patients is sparse, emphasizing the need for case reports to guide management. Hence, we decided to report this case and discuss the risk and mechanisms of splenic injury in a unique subset of patients undergoing endoscopy.

## Case presentation

Presentation

**Figure 1:**
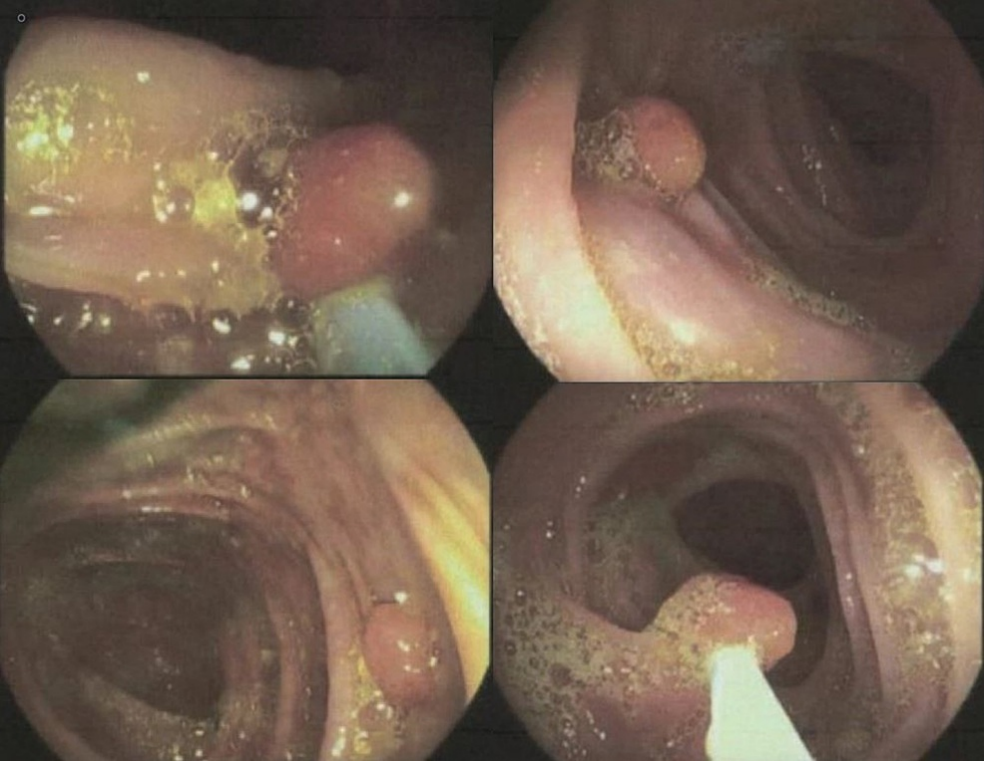
Colonoscopy revealing the identification of polyps in various segments of the colon (top and bottom left) and subsequent snare polypectomy (bottom right).

A 75-year-old male presented to our emergency department (ED) with left upper quadrant abdominal pain, nausea, weakness, and malaise. He had a history of MS, chronic obstructive pulmonary disease (COPD), hypertension, hyperlipidemia, abdominal aortic aneurysm (AAA), transcatheter aortic valve replacement (TAVR), and a bypass for severe peripheral artery disease (PAD). He had a family history significant for MS. He stated that he underwent a routine outpatient colonoscopy the day prior for chronic diarrhea. Colonoscopy revealed three sessile polyps between 0.75 cm-1.25 cm within the transverse colon. The smallest polyp was removed using cold snare polypectomy, while the remaining two larger polyps were removed using hot snare polypectomy. No diverticula were noted (Figure 1). Random colon biopsies were obtained to rule out microscopic and quiescent colitis. On arrival to the ED, he had a blood pressure (BP) - 88/60 mmHg, heart rate - 106 beats/minute, respiratory rate 22 breaths/min, temperature - 34.9°C requiring supplemental oxygen. He appeared ill and frail on physical exam, with a systolic murmur, visible hematomas, and bruising along his extremities. His abdomen was distended and diffusely tender with hypoactive bowel sounds while extremities were pale and cool to touch.

Investigations

**Figure 2:**
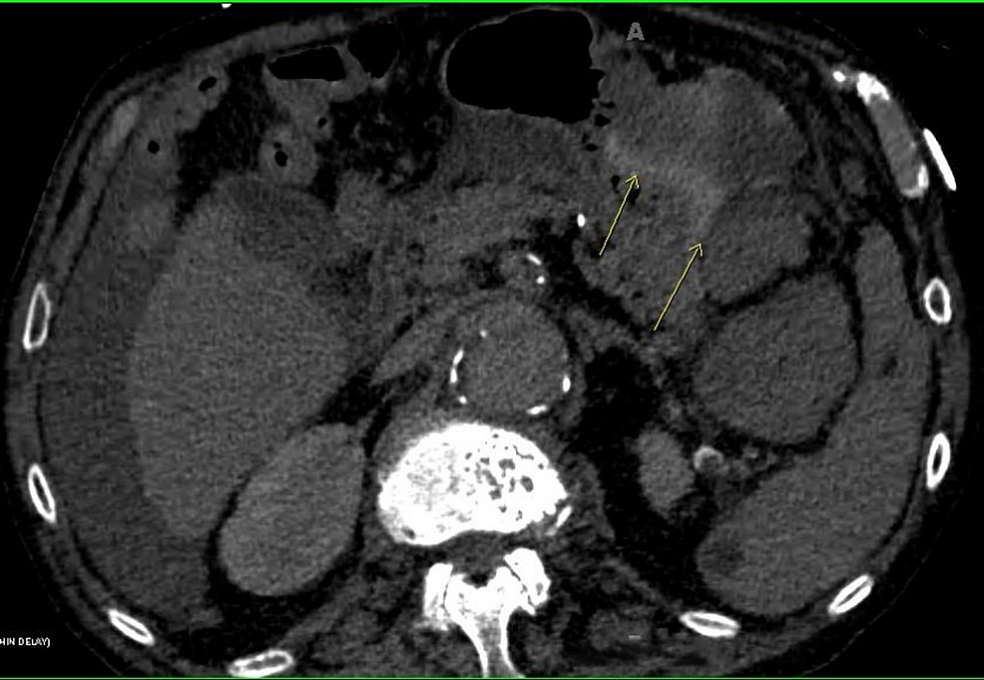
Computed tomography (CT) of the abdomen and pelvis revealed a hematoma in the sub-diaphragm, gastrosplenic ligament, hyper densities around the liver, and bilateral paracolic gutters extending into the pelvis (arrows).

**Figure 3:**
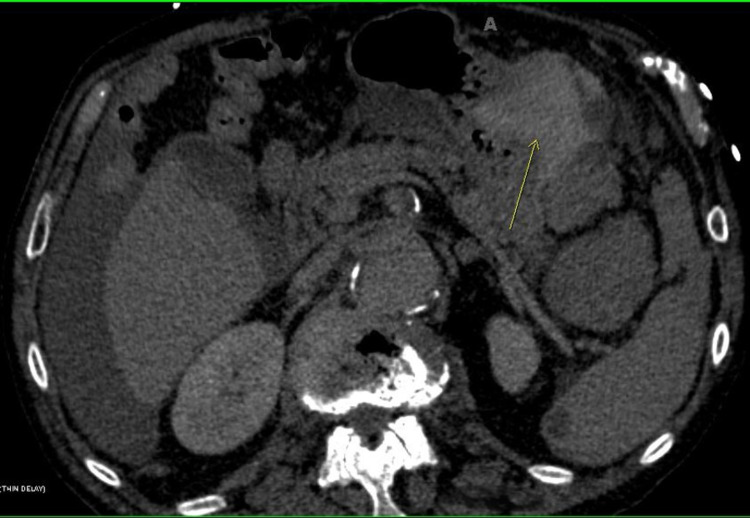
Computed tomography (CT) of the abdomen and pelvis revealed a ruptured splenic capsule (arrow).

Initial investigations revealed a hemoglobin of 6.7 g/dl (baseline 11 g/dl), platelets 62 k/ul, lactic acid 8.92 mg/dl, glucose 200 mg/dl, albumin 2.7 g/dl, and a creatinine of 2.12 mg/dl. Despite aggressive intravenous (IV) resuscitation, his BP remained refractory, requiring vasopressors and a massive transfusion protocol consisting of packed red blood cells (RBC), platelets, and fresh-frozen plasma. He was made nil-per-os (NPO) and started on ceftriaxone and metronidazole. Emergent computed tomography (CT) of the abdomen and pelvis revealed a new hematoma in the left upper quadrant involving the sub-diaphragm, gastrosplenic ligament, with hyper densities around the liver and bilateral paracolic gutters extending into the pelvis (Figures 2, 3). Shortly after, he was taken to the operating room (OR) for an emergency exploratory laparotomy by the surgical service.

Diagnosis

During the exploratory laparotomy, he was found to have a ruptured splenic capsule with an accompanying hematoma that was evacuated with subsequent splenectomy. Adhesions were lysed and no further damage was noted in the abdomen with an intact colon. A total of 2.5-3 liters of abdominal blood was evacuated and the incisions were closed with the placement of a 10 French Blackmore drain into the splenic fossa. His recovery in the surgical intensive care unit (SICU) was uneventful, and he was extubated the following day and subsequently weaned off vasopressors. Drain output remained below 50 ml, and follow-up peritoneal cultures did not reveal any organisms. He was administered pneumococcal and meningococcal vaccines and a prescription for amoxicillin for prophylaxis and discharged to a skilled nursing facility for rehabilitation with a safe transition to home. Follow-up biopsy results from the colonoscopy revealed lymphocytic colitis. He was encouraged to limit non-steroidal anti-inflammatory drugs (NSAIDs) and follow-up for his diarrhea symptoms that eventually subsided without any further therapeutics.

## Discussion

Splenic rupture is a rare yet devastating complication of colonoscopy. A systematic search revealed Ehlers-Danlos syndrome (EDS) to be the most implicated HCTDs in this clinical scenario. This is the first case report of splenic rupture in a patient with known MS from the HCTD cluster to the best of our knowledge. Although vascular EDS has an adverse instrumentation-related perforation rate of 10%, this may be under-reported and potentially less frequent than patients with MS or other variants of EDS [11,12]. As colonoscopy recommendations have changed and awareness has been far greater than before, this exposes numerous patient populations to the procedure. Although our patient had additional risk factors, including surgery and polypectomy, strong consideration should be given to the underlying connective tissue disorder, which may further explain his history of thoracic and abdominal aneurysms that required intervention. A recent study found connective tissue to be significantly associated with perforations amongst a multivariate analysis with more events than non-metastatic cancer, renal, pulmonary, vascular disease, or the ongoing use of anticoagulation [4]. Patients with connective tissue diseases are at a higher risk for instrumentation-related injury, and this case report emphasizes the need for careful selection and pre-endoscopic assessment of these patients.

The mechanism for splenic injury in HCTD has not yet been elucidated; however, it comes in two primary forms: traumatic and atraumatic. Atraumatic mechanisms include: (1) direct trauma when the endoscope crosses the splenic angle or biopsies taken in this location, (2) excessive traction on the splenocolic ligament, or (3) capsular rupture due to traction of splenocolonic adhesions limiting the mobility of the splenic flexure. Using the spleen injury scale, our case fell between grade III and IV [3,13]. Ligament laxity, a core feature of HCTD, can contribute to excessive downward stretching of the splenocolic ligament, stretching, and rupturing blood vessels without tearing the ligament walls [10]. Additionally, splenocolonic adhesions may be more likely in MS due to their propensity to develop accelerated vascular aneurysms requiring frequent surveillance and repairs [9,13]. Although reports of rupture during diagnostic colonoscopies have been reported, polypectomy at the splenic flexure appears to increase the risk of splenic injuries substantially. Our patient initially presented with complaints of chronic diarrhea and underwent a colonoscopy, where multiple random biopsies across the transverse colon were obtained. Simultaneously, colonic histochemical samples from HCTD patients have shown weakened layers from the submucosa until the muscularis propria; thus, these patients are prone to develop inside-out injuries during colonoscopy. Additionally, our patient was frail with significant hypoalbuminemia, reflecting chronic malnutrition, further weakening the tensile strength of these structures.

Strategies to mitigate poor outcomes include a comprehensive assessment beforehand to ensure the appropriateness of the procedure [8]. Alternative non-invasive screening modalities such as fecal immunochemical test (FIT), CT colonography, or Cologuard can be offered to patients who have a strong history of connective tissue disorders or multiple risk factors for vascular fragility [4,11,14]. Intraprocedural care should be taken to limit torque maneuvers used to straighten the colonoscope, prevent loop formation at the splenic flexure, and avoid external manual compression of the left abdomen [11]. Furthermore, HCTD patients should be educated on the concerning signs and symptoms following their colonoscopy, for which they should seek emergent treatment with close, frequent follow-ups [15]. Performing clinicians should have a high index of suspicion for possible splenic complications after colonoscopy, especially in patients with HCTD, as quick recognition and treatment is essential to offset the high morbidity and mortality. Authors suggest prompt surgical evaluation in patients with recent colonoscopy and hemodynamic compromise [3,15]. Urgent laparotomy with splenectomy is recommended when there is active bleeding, hemodynamic instability, or hemoperitoneum, while a laparoscopic approach can be first-line in stable patients [5,13,16]. High-risk patients can be offered coil embolization of the splenic artery [17]. There are no existing guidelines to determine which HCTD patients are at higher risk for splenic injury after colonoscopy. Predictors of aortic dissection like aortic diameter and family history are used infrequently, although it has not been validated [9].

Limitations of the available studies include cohort or population studies that may have had MS cases, although they were not captured in our review. Additionally, within MS, another rare entity involving the spleen exists, known as “wandering spleen,” characterized by ligament weakness and the propensity to avulse itself. Congenital and acquired cases of wandering spleen are more frequently seen in the pediatric age group. Our patient had multiple risk factors, including extensive surgical history, history of bowel disease, and anticoagulant use, placing him at high procedural risk as well.

Future studies or risk-scoring assessment tools that incorporate connective tissue disorders are needed to improve patient selection. HCTD patients pose technical (intubation and withdrawal) challenges due to increased tissue laxity and colonic distensibility, which may be improved by using colonoscopies with machine learning algorithms that have improved dexterity and stabilization and may be beneficial in patients with fragile or complicated anatomy [10,18].

## Conclusions

As the population undergoes frequent endoscopic assessments for gastrointestinal etiologies, specific at-risk populations that are missed in population studies are patients with HCTDs or connective tissue diseases. The existing literature demonstrates HCTD to be a significant risk factor, higher than other systemic illnesses or use of ongoing anticoagulation. These patients are at risk of instrumentation-related injuries due to ligament laxities, fragile mucosa, and complicated anatomies. Amongst these, splenic injuries are exceedingly rare, yet they carry a high mortality. A careful history and physical examination are essential in patient selection, and other modalities of CRC screening may be considered, such as FIT, occult testing, CT colonography, or Cologuard.
